# Supplementary material for: Ontological Analysis of Coronavirus Associated Human Genes at the COVID-19 Disease Portal
Source: Genes (Basel). 2022 Dec 7;13(12):2304. doi: 10.3390/genes13122304 (PMC9777590; doi:10.3390/genes13122304)
Supplement: Supplementary file 1 [file genes-13-02304-s001.zip › genes-2055809-supplementary.pdf]

Figure S1

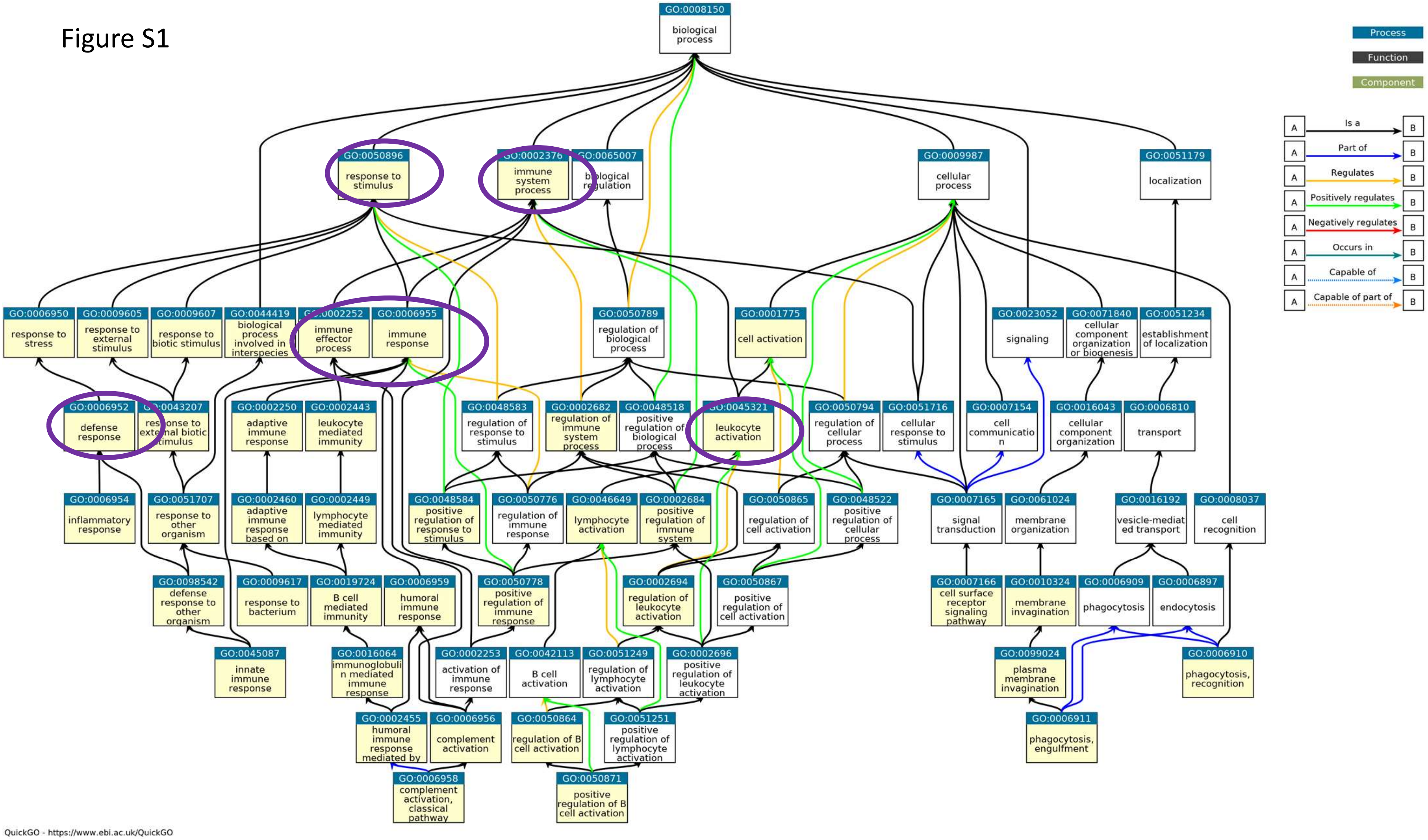
GO:0008150 biological process
Process
Function
Component
Is a
Part of
Regulates
Positively regulates
Negatively regulates
Occurs in
Capable of
Capable of part of
GO:0050896 response to stimulus
GO:0002376 immune system process
GO:0065007 biological regulation
GO:0009987 cellular process
GO:0051179 localization
GO:0006950 response to stress
GO:0009605 response to external stimulus
GO:0009607 response to biotic stimulus
GO:0044419 biological process involved in interspecies
GO:0002252 immune effector process
GO:0006955 immune response
GO:0050789 regulation of biological process
GO:0001775 cell activation
GO:0023052 signaling
GO:0071840 cellular component organization or biogenesis
GO:0051234 establishment of localization
GO:0006952 defense response
GO:0043207 response to external biotic stimulus
GO:0002250 adaptive immune response
GO:0002443 leukocyte mediated immunity
GO:0048583 regulation of response to stimulus
GO:0002682 regulation of immune system process
GO:0048518 positive regulation of biological process
GO:0045321 leukocyte activation
GO:0050794 regulation of cellular process
GO:0051716 cellular response to stimulus
GO:0007154 cell communication
GO:0016043 cellular component organization
GO:0006810 transport
GO:0006954 inflammatory response
GO:0051707 response to other organism
GO:0002460 adaptive immune response based on
GO:0002449 lymphocyte mediated immunity
GO:0048584 positive regulation of response to stimulus
GO:0050776 regulation of immune response
GO:0046649 lymphocyte activation
GO:0002684 positive regulation of immune system
GO:0050865 regulation of cell activation
GO:0048522 positive regulation of cellular process
GO:0007165 signal transduction
GO:0061024 membrane organization
GO:0016192 vesicle-mediated transport
GO:0008037 cell recognition
GO:0098542 defense response to other organism
GO:0009617 response to bacterium
GO:0019724 B cell mediated immunity
GO:0006959 humoral immune response
GO:0050778 positive regulation of immune response
GO:0002694 regulation of leukocyte activation
GO:0050867 positive regulation of cell activation
GO:0007166 cell surface receptor signaling pathway
GO:0010324 membrane invagination
GO:0006909 phagocytosis
GO:0006897 endocytosis
GO:0045087 innate immune response
GO:0016064 immunoglobulin mediated immune response
GO:0002253 activation of immune response
GO:0042113 B cell activation
GO:0051249 regulation of lymphocyte activation
GO:0002696 positive regulation of leukocyte activation
GO:0099024 plasma membrane invagination
GO:0006910 phagocytosis, recognition
GO:0002455 humoral immune response mediated by
GO:0006956 complement activation
GO:0050864 regulation of B cell activation
GO:0051251 positive regulation of lymphocyte activation
GO:0006911 phagocytosis, engulfment
GO:0006958 complement activation, classical pathway
GO:0050871 positive regulation of B cell activation
QuickGO - https://www.ebi.ac.uk/QuickGO

Figure S1. The Gene Ontology (GO) Ancestor chart showing the relations among the enriched biological process (BP) GO terms associated with COVID-19 disease genes. The enriched GO terms are highlighted in yellow, and the circled terms are enriched high level BP terms. ‘response to stimulus’ and ‘immune system process’ are the two major branches where most of the enriched terms reside.

Figure S2

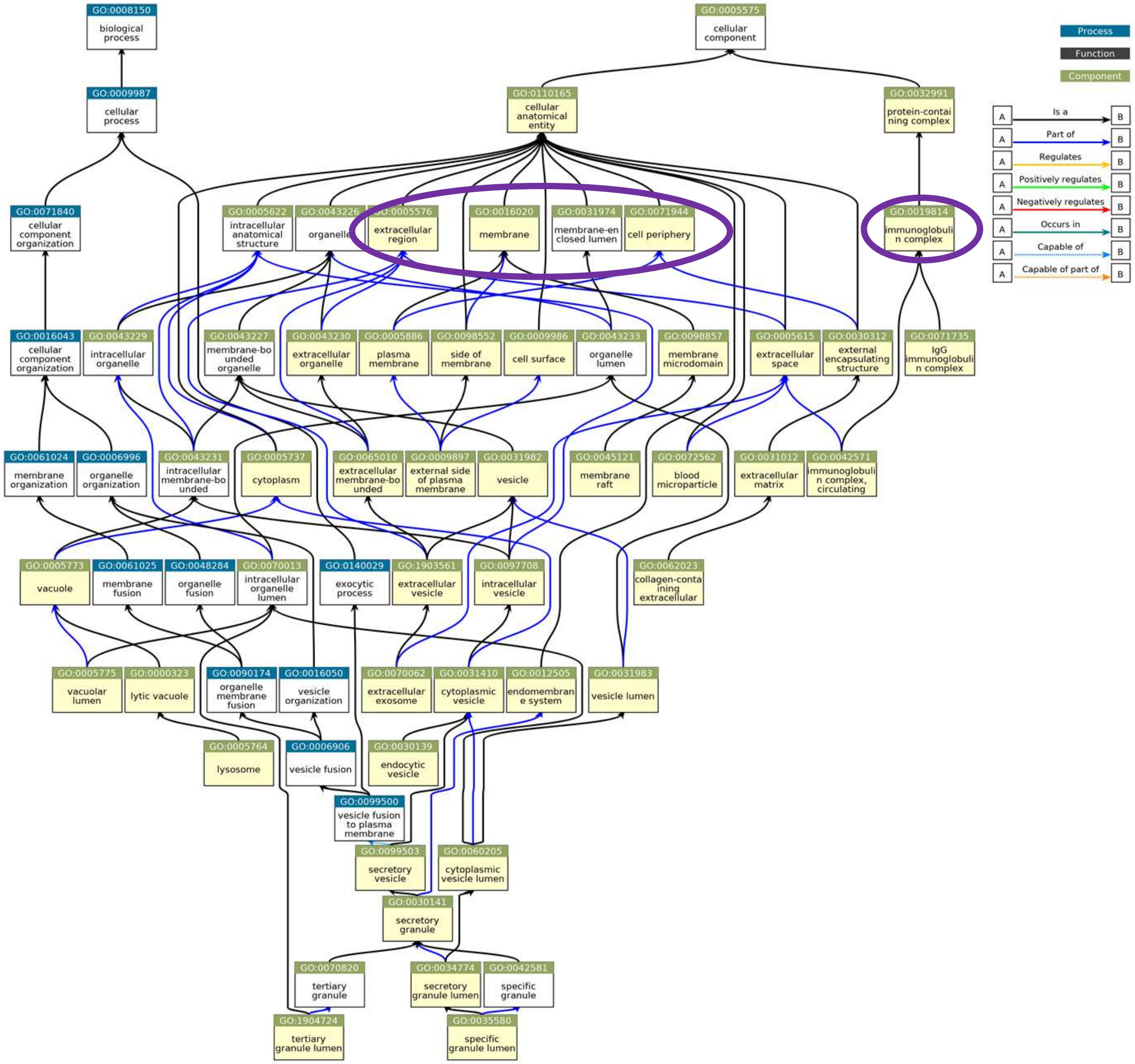

QuickGO - https://www.ebi.ac.uk/QuickGO

Figure S2. The Gene Ontology (GO) Ancestor chart showing the relations among the enriched cellular component (CC) GO terms associated with COVID-19 disease genes. The enriched GO terms are highlighted in yellow, and the circled terms are enriched high level CC terms.

Figure S3

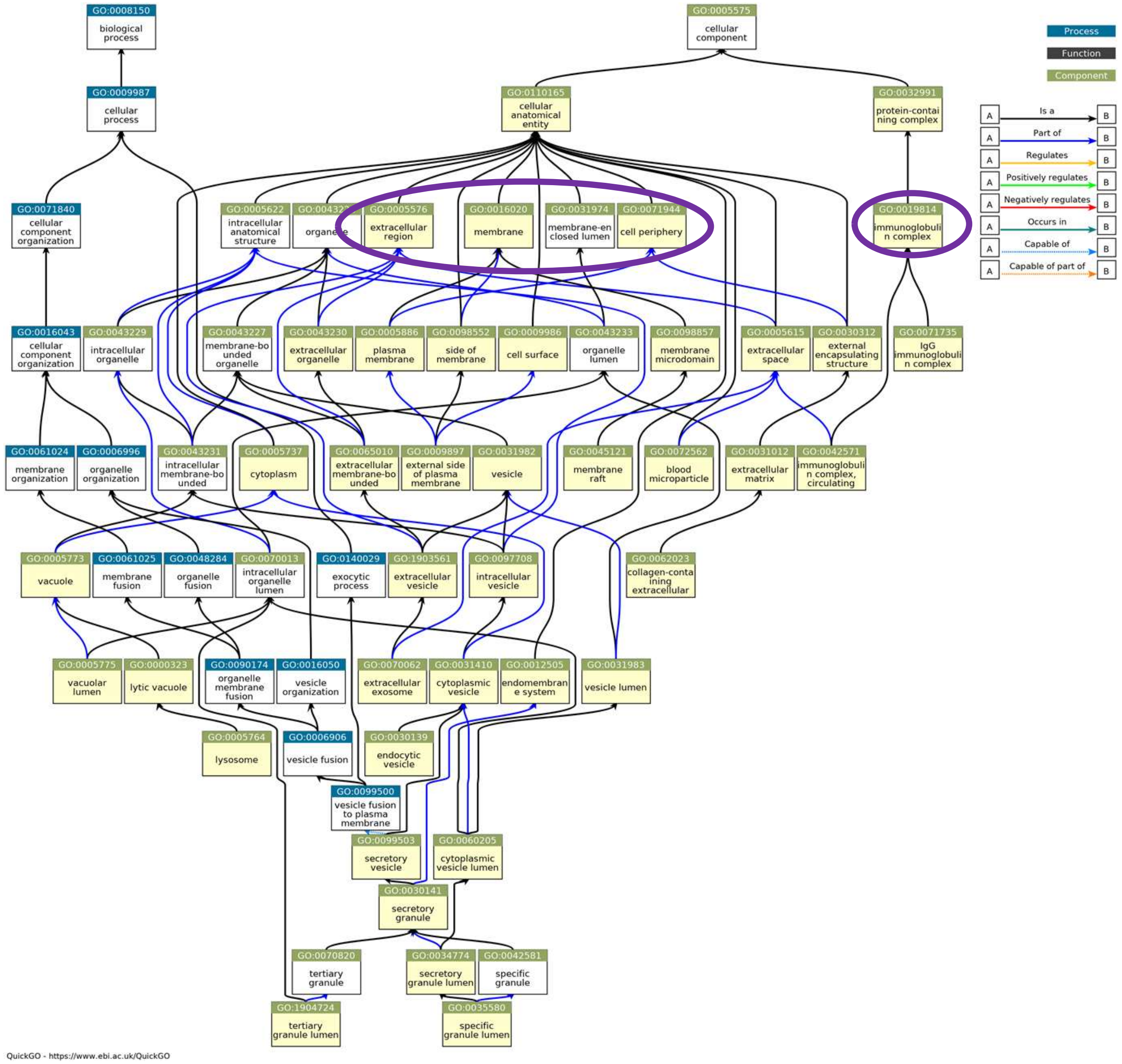
GO:0008150
biological process
GO:0009987
cellular process
GO:0005575
cellular component
GO:0110165
cellular anatomical entity
GO:0032991
protein-containing complex
Process
Function
Component
Is a
Part of
Regulates
Positively regulates
Negatively regulates
Occurs in
Capable of
Capable of part of
GO:0071840
cellular component organization
GO:0005622
intracellular anatomical structure
organelle
GO:0005576
extracellular region
GO:0016020
membrane
GO:0031974
membrane-enclosed lumen
GO:0071944
cell periphery
GO:0019814
immunoglobulin complex
GO:0016043
cellular component organization
GO:0043229
intracellular organelle
GO:0043227
membrane-bounded organelle
GO:0043230
extracellular organelle
GO:0005886
plasma membrane
GO:0098552
side of membrane
GO:0009986
cell surface
GO:0043233
organelle lumen
GO:0098857
membrane microdomain
GO:0005615
extracellular space
GO:0030312
external encapsulating structure
GO:0071735
IgG immunoglobulin complex
GO:0061024
membrane organization
GO:0006996
organelle organization
GO:0043231
intracellular membrane-bounded
GO:0005737
cytoplasm
GO:0065010
extracellular membrane-bounded
GO:0009897
external side of plasma membrane
GO:0031982
vesicle
GO:0045121
membrane raft
GO:0072562
blood microparticle
GO:0031012
extracellular matrix
GO:0042571
immunoglobulin complex, circulating
GO:0005773
vacuole
GO:0061025
membrane fusion
GO:0048284
organelle fusion
GO:0070013
intracellular organelle lumen
GO:0140029
exocytic process
GO:1903561
extracellular vesicle
GO:0097708
intracellular vesicle
GO:0062023
collagen-containing extracellular
GO:0005775
vacuolar lumen
GO:0000323
lytic vacuole
GO:0090174
organelle membrane fusion
GO:0016050
vesicle organization
GO:0070062
extracellular exosome
GO:0031410
cytoplasmic vesicle
GO:0012505
endomembrane system
GO:0031983
vesicle lumen
GO:0005764
lysosome
GO:0006906
vesicle fusion
GO:0030139
endocytic vesicle
GO:0099500
vesicle fusion to plasma membrane
GO:0099503
secretory vesicle
GO:0060205
cytoplasmic vesicle lumen
GO:0030141
secretory granule
GO:0070820
tertiary granule
GO:0034774
secretory granule lumen
GO:0042581
specific granule
GO:1904724
tertiary granule lumen
GO:0035580
specific granule lumen
QuickGO - https://www.ebi.ac.uk/QuickGO

Figure S3. The Gene Ontology (GO) Ancestor chart showing the relations among the enriched molecular function (MF) GO terms associated with COVID-19 disease genes. The enriched GO terms are highlighted in yellow, and the circled terms are enriched high level BP terms. ‘molecular function regulator’ and ‘binding’ are the two major branches where most of the enriched terms reside.
